# Risk of chronic pancreatitis in carriers of loss-of-function *CTRC* variants: A meta-analysis

**DOI:** 10.1371/journal.pone.0268859

**Published:** 2022-05-20

**Authors:** Amanda Takáts, Gergő Berke, Noémi Gede, Balázs Csaba Németh, Heiko Witt, Stanisław Głuszek, Agnieszka Magdalena Rygiel, Péter Hegyi, Miklós Sahin-Tóth, Eszter Hegyi

**Affiliations:** 1 Institute for Translational Medicine, Medical School, University of Pécs, Pécs, Hungary; 2 Department of Medicine, Albert Szent-Györgyi Medical School, University of Szeged, Szeged, Hungary; 3 Pediatric Nutritional Medicine & Else Kröner-Fresenius-Centre for Nutritional Medicine (EKFZ), Technical University Munich (TUM), Munich, Germany; 4 Collegium Medicum Jan Kochanowski University, Kielce, Poland; 5 Department of Medical Genetics, Institute of Mother and Child, Warsaw, Poland; 6 Centre for Translational Medicine, Semmelweis University, Budapest, Hungary; 7 Department of Surgery, University of California Los Angeles, Los Angeles, California, United States of America; Odense University Hospital, DENMARK

## Abstract

The digestive protease chymotrypsin C (CTRC) protects the pancreas against pancreatitis by degrading potentially harmful trypsinogen. Loss-of-function genetic variants in *CTRC* increase risk for chronic pancreatitis (CP) with variable effect size, as judged by the reported odds ratio (OR) values. Here, we performed a meta-analysis of published studies on four variants that alter the CTRC amino-acid sequence, are clinically relatively common (global carrier frequency in CP >1%), reproducibly showed association with CP and their loss of function phenotype was verified experimentally. We found strong enrichment of *CTRC* variants p.A73T, p.V235I, p.K247_R254del, and p.R245W in CP cases versus controls, yielding OR values of 6.5 (95% confidence interval (CI) 2.4–17.8), 4.5 (CI 2.2–9.1), 5.4 (CI 2.6–11.0), and 2.6 (CI 1.6–4.2), respectively. Subgroup analysis demonstrated disease association of variants p.K247_R254del and p.R245W in alcoholic CP with similar effect sizes as seen in the overall CP group. Homozygosity or compound heterozygosity were rare and seemed to be associated with higher risk. We also identified a so far unreported linkage disequilibrium between variant p.K247_R254del and the common c.180C>T (p.G60 =) haplotype. Taken together, the results indicate that heterozygous loss-of-function *CTRC* variants increase the risk for CP approximately 3-7-fold. This meta-analysis confirms the clinical significance of *CTRC* variants and provides further justification for the genetic screening of CP patients.

## Introduction

Chronic pancreatitis (CP) is a progressive fibro-inflammatory disorder of the pancreas that often develops in the background of genetic predisposition [1]. A number of susceptibility genes have been identified and many of these have been shown to influence the activation of the digestive protease precursor trypsinogen to its active form trypsin [2]. Pathological, intrapancreatic activation of trypsinogen can occur through autoactivation, a reaction in which trypsin activates trypsinogen. Defense mechanisms that protect the pancreas against trypsinogen autoactivation and trypsin activity include the serine protease inhibitor Kazal type 1 (SPINK1) and chymotrypsin C (CTRC), which can readily degrade trypsinogen and thereby suppress its activation [2–4]. Gain-of-function mutations in the serine protease 1 (*PRSS1*) gene encoding human cationic trypsinogen and loss-of-function mutations in the *SPINK1* and *CTRC* genes stimulate autoactivation of trypsinogen and increase the risk for CP [2]. The most common *PRSS1* mutations exert their stimulatory effect on autoactivation by blocking CTRC-dependent trypsinogen degradation [2, 5]. Genetic changes can be also protective, as exemplified by the p.G191R variant in the *PRSS2* gene encoding human anionic trypsinogen [6]. This variant causes autodegradation of anionic trypsinogen and thereby decreases the risk for CP. Similarly, a common inversion at the chymotrypsin B1-B2 (*CTRB1-CTRB2*) locus increases the expression of CTRB2, which leads to more effective degradation of anionic trypsinogen and reduced CP risk [7].

*CTRC* as a pancreatitis risk gene was identified in 2008 [4, 8] and studies to date have described a large number of missense mutations and a microdeletion found in CP cases [9–20, see also www.pancreasgenetics.org]. The majority of these variants were detected in a few cases only and only four variants were found to associate with CP in a statistically significant manner: Variants c.738_761del24 (p.K247_R254del) and c.760C>T (p.R254W) were found primarily in European cohorts with approximate carrier frequencies of 1–2% while variants c.217G>A (p.A73T) and c.703G>A (p.V235I) were detected in Indian cohorts with carrier frequencies of 1–5%. As judged by the odds ratio (OR) values, the effect sizes reported for these CTRC variants were variable, mostly in the range of 3-10-fold, however, a lack of effect and an OR of 19 were also described. Functional studies demonstrated that these four variants caused loss of CTRC function by various mechanisms that included reduced secretion from cells, decreased catalytic activity and increased sensitivity to degradation by trypsin [4, 21, 22]. Furthermore, variant p.A73T was shown to induce endoplasmic reticulum (ER) stress [21, 22]. Similar functional defects were observed with many of the rare CTRC variants [4, 21–23].

In addition to the low-frequency missense and microdeletion variants, a common haplotype (circa 30% carrier frequency in CP cases) consisting of the c.180C>T (p.G60 =) variant in exon 3 and the c.493+52G>A variant in intron 5, was found reproducibly to increase CP risk by about 2-fold in the heterozygous state and perhaps as much as 10-fold in homozygous carriers [7–11, 13, 17]. The functional impact and the disease-relevant variant within the haplotype remain unclear; however, reduced CTRC expression possibly due to altered pre-mRNA splicing was hypothesized [2]. Unlike the low-frequency missense and microdeletion CTRC variants, the p.G60 = haplotype variants do not alter the amino-acid sequence of CTRC and cannot cause enzyme activity changes by any of the mechanisms described for the other variants.

The aim of the present study was to determine the global effect size of missense *CTRC* variants on CP risk, by conducting a meta-analysis of all published genetic association studies. We focused our efforts on the four low-frequency missense and microdeletion variants, as the limited information on the rare and private missense *CTRC* variants precluded a formal analysis. Establishing the precise effect size of *CTRC* variants on CP risk has important implications for genetic testing of CP patients and counseling of *CTRC* variant carriers.

## Methods

### Study design

In this meta-analysis, we set out to evaluate the risk effect of CTRC variants that (i) alter the amino-acid sequence of CTRC, (ii) are relatively frequent (>1% carrier frequency in CP cases), (iii) showed reproducible association with CP at least in 2 independent cohorts, and (iv) were demonstrated to cause a loss of CTRC function experimentally.

#### Protocol registration

The present work is reported in accordance with the Preferred Reporting Items for Systematic Reviews and Meta-Analyses (PRISMA) Statement (S1 Table) [24]. The protocol of the meta-analysis was registered in advance in the PROSPERO database under the registration number CRD42018111537.

### Search strategy

We performed a systematic search on April 5, 2022, in three databases (MEDLINE via PubMed Central, Embase, and Cochrane Central Register of Controlled Trials) using the following search keys: pancreatitis AND (CTRC OR “Chymotrypsin C” OR Caldecrin OR”Elastase 4” OR ELA4 OR CLCR) AND (polymorphism OR polymorphisms OR variant OR variants OR mutation OR mutations) OR “p.A73T” OR “p.V235I” OR “p.K247_R254del” OR “p.R254W”. No filters were applied. As an additional data source, we used the www.pancreasgenetics.org database of genetic risk factors.

### Study selection and data extraction

Genetic association case-control studies investigating the following low-frequency *CTRC* variants were included: p.A73T, p.V235I, p.K247_254del, and p.R254W. Articles identified by the initial search were imported into a reference management program (EndNoteX7.4; Clarivate Analytics, Philadelphia, PA). After removing overlaps between databases and duplicate references, the remaining records were screened by title, abstract and full text by two authors independently. Disagreements were resolved by the corresponding author. Eligible original studies were subjected to data collection onto a pre-defined Excel sheet. The following data were extracted: first author, publication year, basic demographics (ethnicity, age), type of pancreatitis, number of cases and controls, carrier frequencies of the *CTRC* variants analyzed in this study, and type of the genetic screening method. In cases of suspected overlap between study populations, studies with the highest number of participants were included in the final analysis. Zero event studies were excluded from the statistical analysis.

### Quality assessment

A modified version of the Newcastle-Ottawa Scale (NOS) was used for the quality assessment of the included case-control studies [25].

### Statistical analysis

The effect of the different *CTRC* variants was assessed by calculating pooled odds ratios (OR) with 95% confidence intervals (CI). Random-effects model with Der-Simonian Laird estimation was applied. Heterogeneity was examined with the I^2^-test (p≥0.1). Sensitivity analyses (leave-one-out method) were also performed. Publication bias was ruled out by visual inspection of funnel plots and by Egger’s test. Statistical analyses were performed with Stata 15 (Stata Corp).

## Results

### Study design

**Fig 1:**
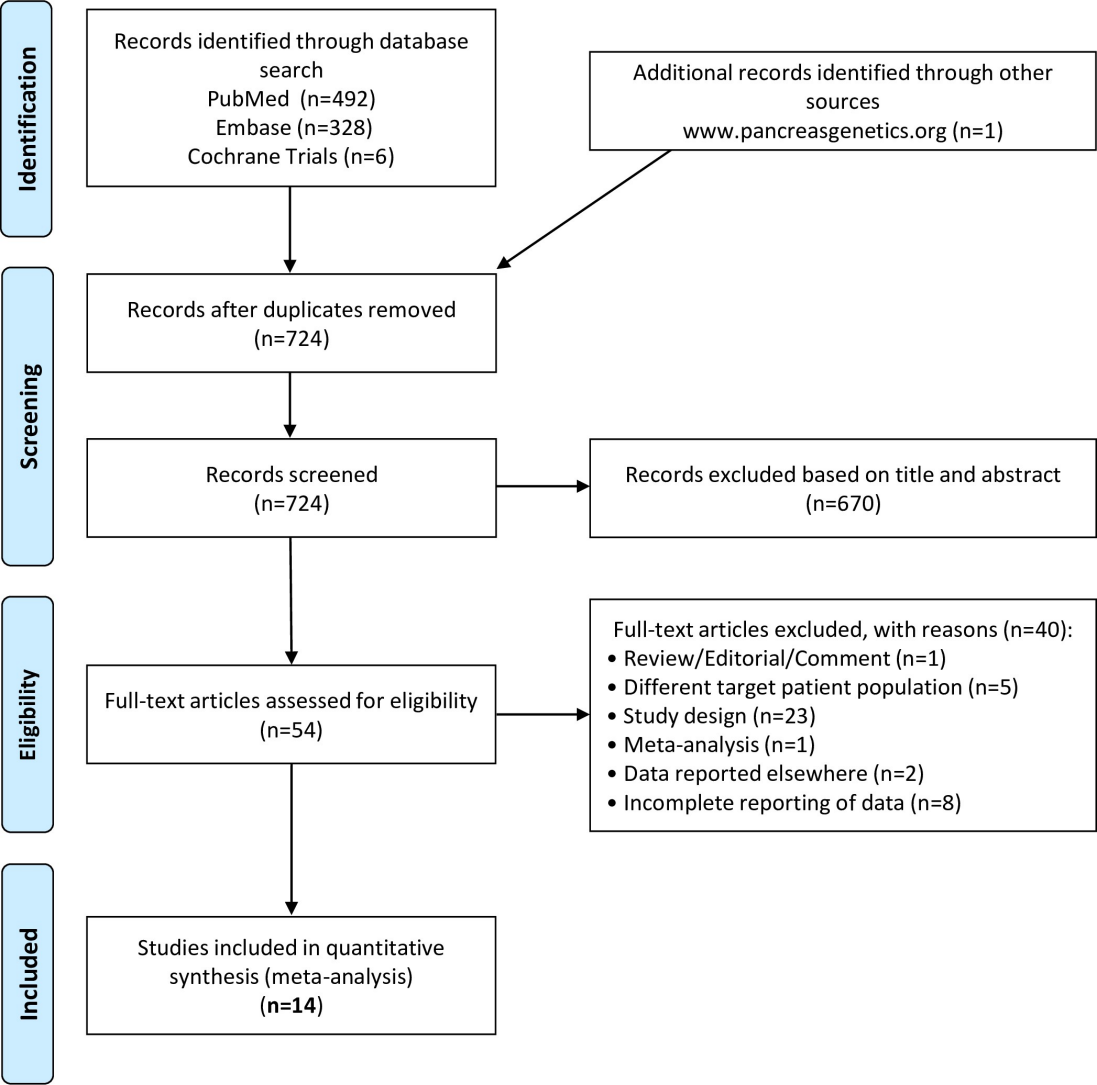
PRISMA flow diagram showing the systematic search and selection process.

We found four variants that met our inclusion criteria, three missense mutations and a microdeletion: p.A73T, p.V235I, p.K247_R254del, and p.R254W. We performed a comprehensive database search and retrieved all published case-control studies that included any of these variants. Fig 1 demonstrates the protocol employed for study selection, which resulted in the identification of 14 articles [4, 8–20] suitable for inclusion in this meta-analysis (Table 1).

**Table 1:** Characteristics of the studies included in the meta-analysis.

Study	Ethnicity	Study cohort	Etiology	Control population	Detected variants	Screening method
		(n)	(n)	(n)		
Rosendahl et al., 2008	German	CP (1249)	HP (143)	Healthy (2804)	p.V235I, p.K247_R254del, p.R254W	Sanger sequencing of all exons
			ICP (758)			
			ACP (348)	ALD (432)		
	Indian	CP (71)	TCP (71)	Healthy (84)	p.A73T, p.V235I, p.R254W	
Masson et al., 2008	French white	CP (287)	HP (29)	Healthy (350)	p.A73T, p.V235I, p.K247_R254del, p.R254W	Sanger sequencing of all exons in patients; Sanger sequencing of all exons or DHPLC in controls
			FCP (42)			
			ICP (216)			
Derikx et al., 2009	Indian	CP (150)	TCP (150)	ND (150)	p.A73T, p.V235I	Sanger sequencing of all exons
Paliwal et al., 2013	Indian	CP (584)	ICP (87)	No pancreatitis (598)	p.A73T, p.V235I, p.R254W	Sanger sequencing of all exons in patients and 230 controls; targeted screening in remaining controls
			TCP (497)			
Masamune et al., 2013	Japanese	CP (506)	HP (35)	Healthy (274)	p.R254W	Sanger sequencing of all exons
			FCP (21)			
			ICP (206)			
			ACP (244)			
Schubert et al., 2014	German, Russian	CP (102)	ICP (102)	Healthy (130)	p.R254W	Sanger sequencing of exon 7
LaRusch et al., 2015*	USA (European ancestry)	RAP (448)	ARAP (109)	Healthy (1017)	p.A73T, p.K247_R254del, p.R254W	Sanger sequencing of exons 2–3 and 7; RFLP genotyping for p.K247_R254del, p.R254W
			NARAP (339)			
		CP (694)	ACP (284)			
			NACP (410)			
Koziel et al., 2015	Polish	RAP (82)	ARAP (ND)	Healthy (345)	p.V235I, p.K247_R254del	HRM-PCR and Sanger sequencing of exons 3 and 7
			NARAP (ND)			
Sofia et al., 2016	Italian	CP (80)	ICP (80)	Healthy (50)	p.V235I	NGS
Costa et al., 2016	Brazilian (white/African/Asian)	CP (148)	ICP (38)	Healthy (297)	p.R254W	Sanger sequencing of exon 7
			ACP (110)	Chronic alcoholics (110)		
Grabarczyk et al., 2017*	Polish	Pediatric CP (136)	ICP (61)	Healthy (401)	p.K247_R254del, p.R254W	Sanger sequencing of exons 2–7 in patients; HRM-PCR and Sanger sequencing in controls
			Other risk factor present** (75)			
Phillips et al., 2018	African-American	RAP (45)	ND (45)	ND (238)	p.R254W	PCR-RFLP for p.K247_R254del and p.R254W, Sanger sequencing
		CP (232)	ND (232)			
Zou et al., 2018	Han Chinese	CP (1061)	ICP (715)	Healthy (1196)	p.V235I, p.R254W	NGS
			ACP (206)			
			SCP (140)			
Cichoż-Lach et al., 2019	Polish	CP (176)	ACP (124)	Healthy (52)	p.K247_R254del, p.R254W	PCR-RFLP for p.R254W and p.K247_R254del, Sanger sequencing
			NACP (52)			

CP, chronic pancreatitis; RAP, recurrent acute pancreatitis; HP, hereditary pancreatitis; ICP, idiopathic chronic pancreatitis; ACP, alcoholic chronic pancreatitis; TCP, tropical chronic pancreatitis; FCP, familial chronic pancreatitis; NACP, non-alcoholic chronic pancreatitis; ARAP, alcoholic recurrent acute pancreatitis; NARAP, non-alcoholic recurrent acute pancreatitis; SCP, smoking-associated chronic pancreatitis; ALD, alcoholic liver disease; DHPLC, denaturing high performance liquid chromatography; RFLP, restriction fragment length polymorphism; NGS, next-generation sequencing; PCR, polymerase chain reaction; HRM-PCR, high-resolution melting PCR; ND, not determined.

*Please see the referenced article for the exact number of cases and controls for each variant.

**Gene mutations, anatomic anomalies, metabolic, biliary, or autoimmune disorders.

All four *CTRC* variants were detected worldwide, however, with uneven geographic/ethnic distribution. Thus, variants p.A73T and p.V235I were most frequently reported in South-Asian cohorts whereas variants p.K247_R254del and p.R254W were typically found in subjects of European origin. Considering the relatively small number of studies available, we chose to perform a global meta-analysis of the selected *CTRC* variants without regional or ethnic subgrouping.

In our analysis, we included both alcoholic CP and non-alcoholic CP cohorts, such as idiopathic, hereditary, familial and tropical CP. If reported separately, recurrent acute pancreatitis (RAP) cases were pooled with CP cases, while acute pancreatitis cases were excluded. Due to the somewhat inconsistent classification and/or reporting, subgroup analysis of the various CP cohorts was feasible only for alcoholic CP. Different CP cohorts (e.g. alcoholic and non-alcoholic) from a given publication were combined if these were compared with the same control group. Alcoholic and tropical CP cohorts from certain studies were treated separately (labeled ACP and TCP in the figures) if distinct control groups were used for comparison.

### Association analysis

**Fig 2:**
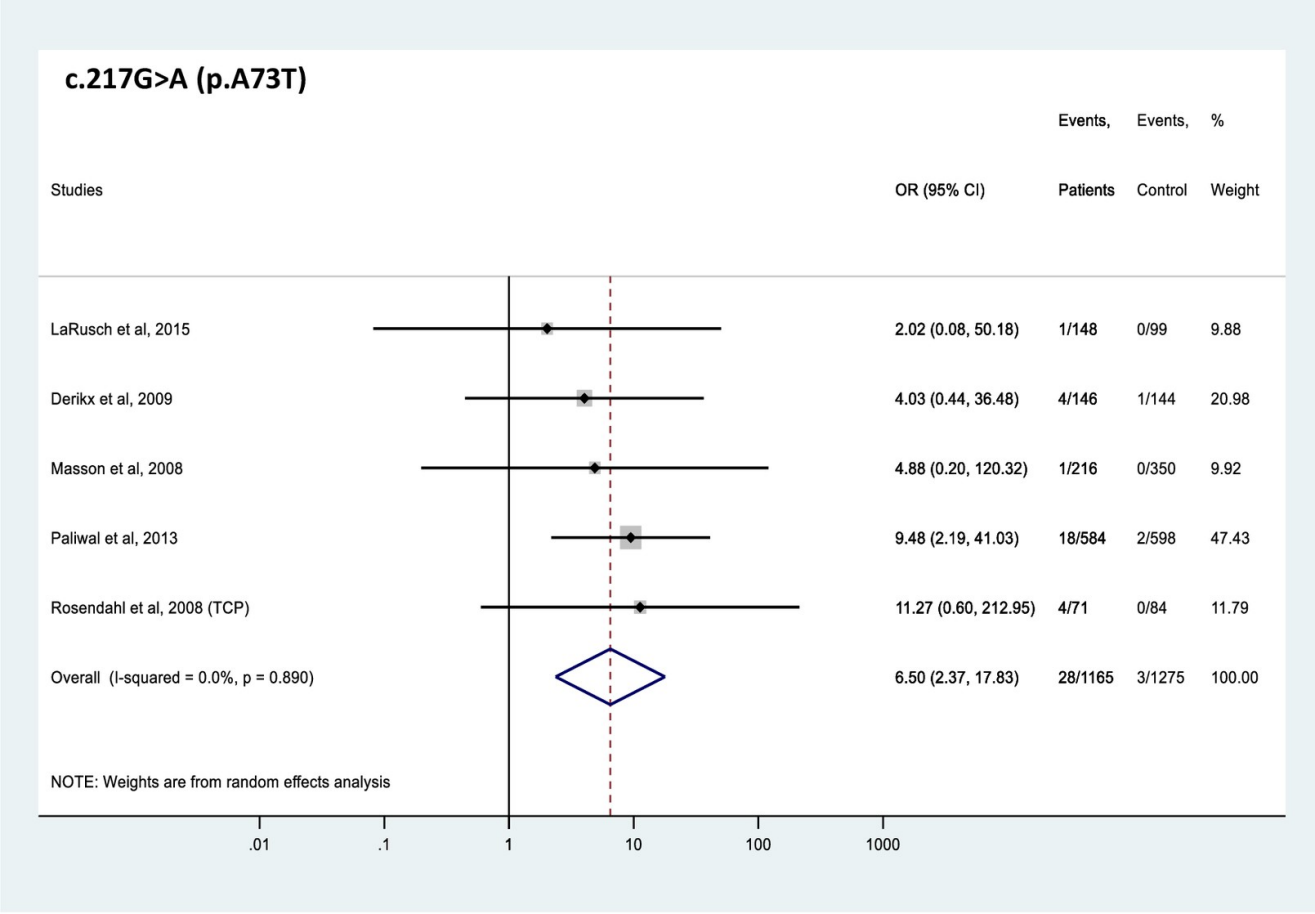
Forest plot showing odds ratios for pancreatitis risk in patients carrying the c.217G>A (p.A73T) *CTRC* variant. OR, odds ratio; CI, confidence interval.

**Fig 3:**
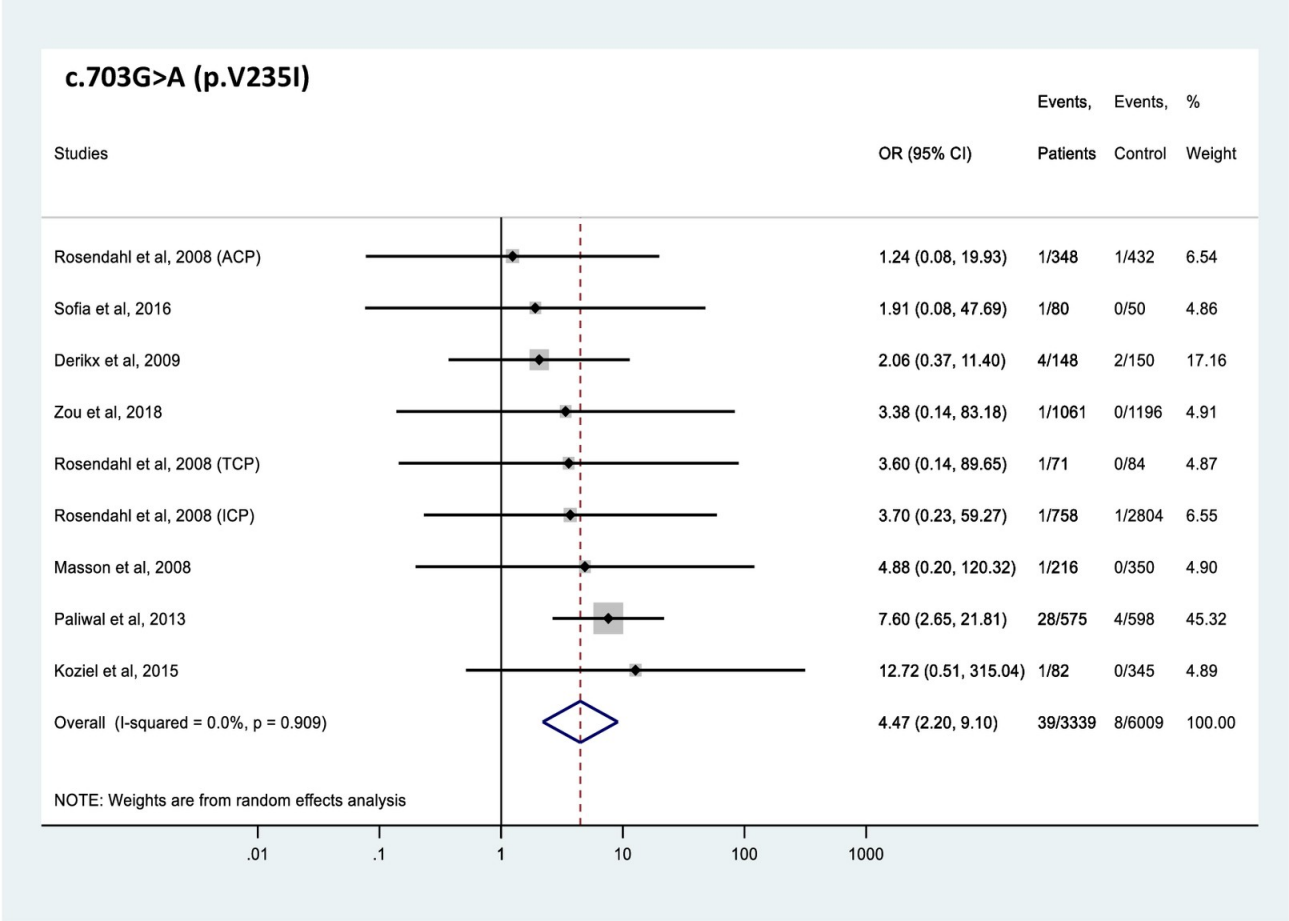
Forest plot showing odds ratios for pancreatitis risk in patients carrying the c.703G>A (p.V235I) *CTRC* variant. OR, odds ratio; CI, confidence interval.

**Fig 4:**
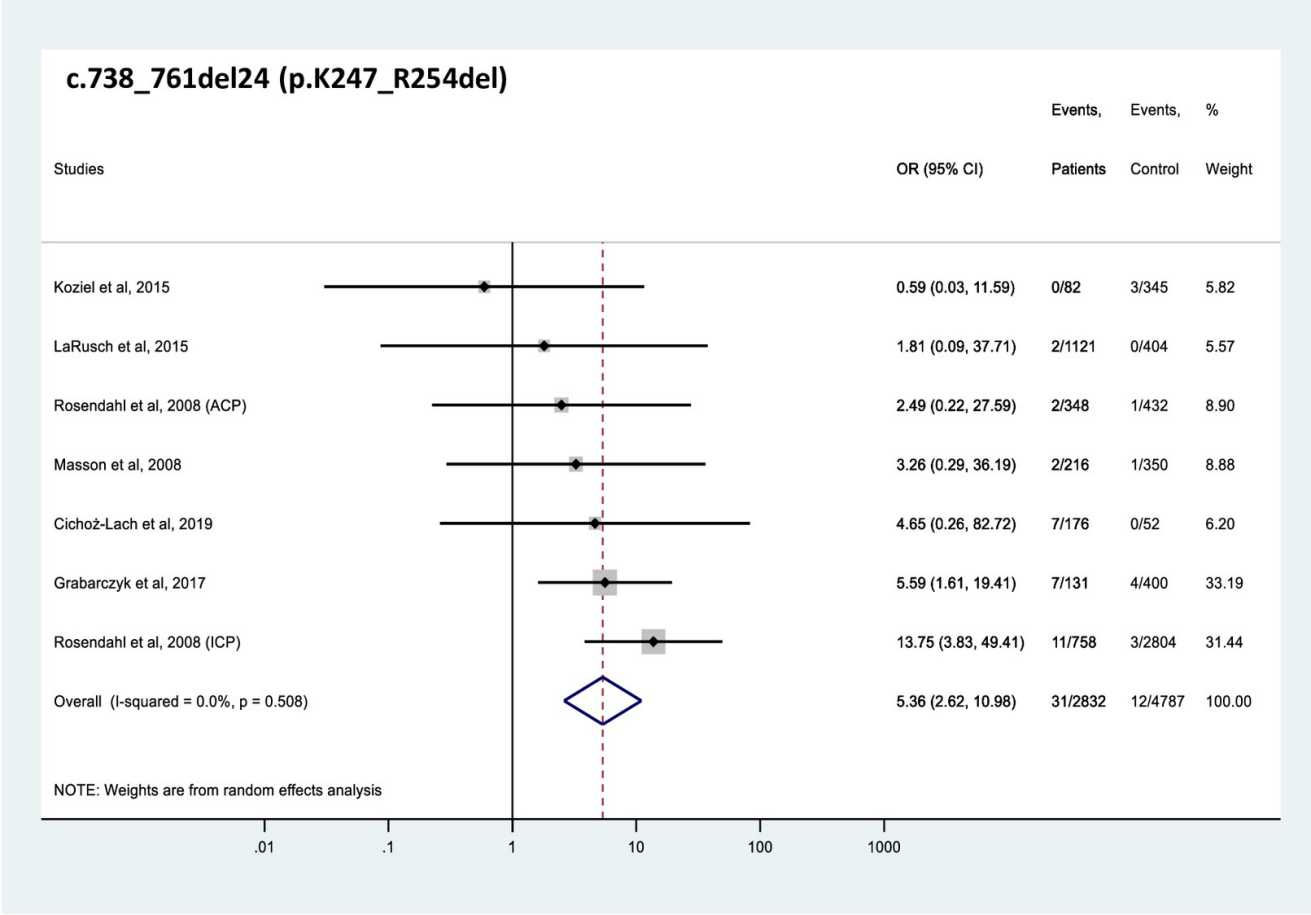
Forest plot showing odds ratios for pancreatitis risk in patients carrying the c.738_761del24 (p.K247_R254del) *CTRC* variant. OR, odds ratio; CI, confidence interval.

**Fig 5:**
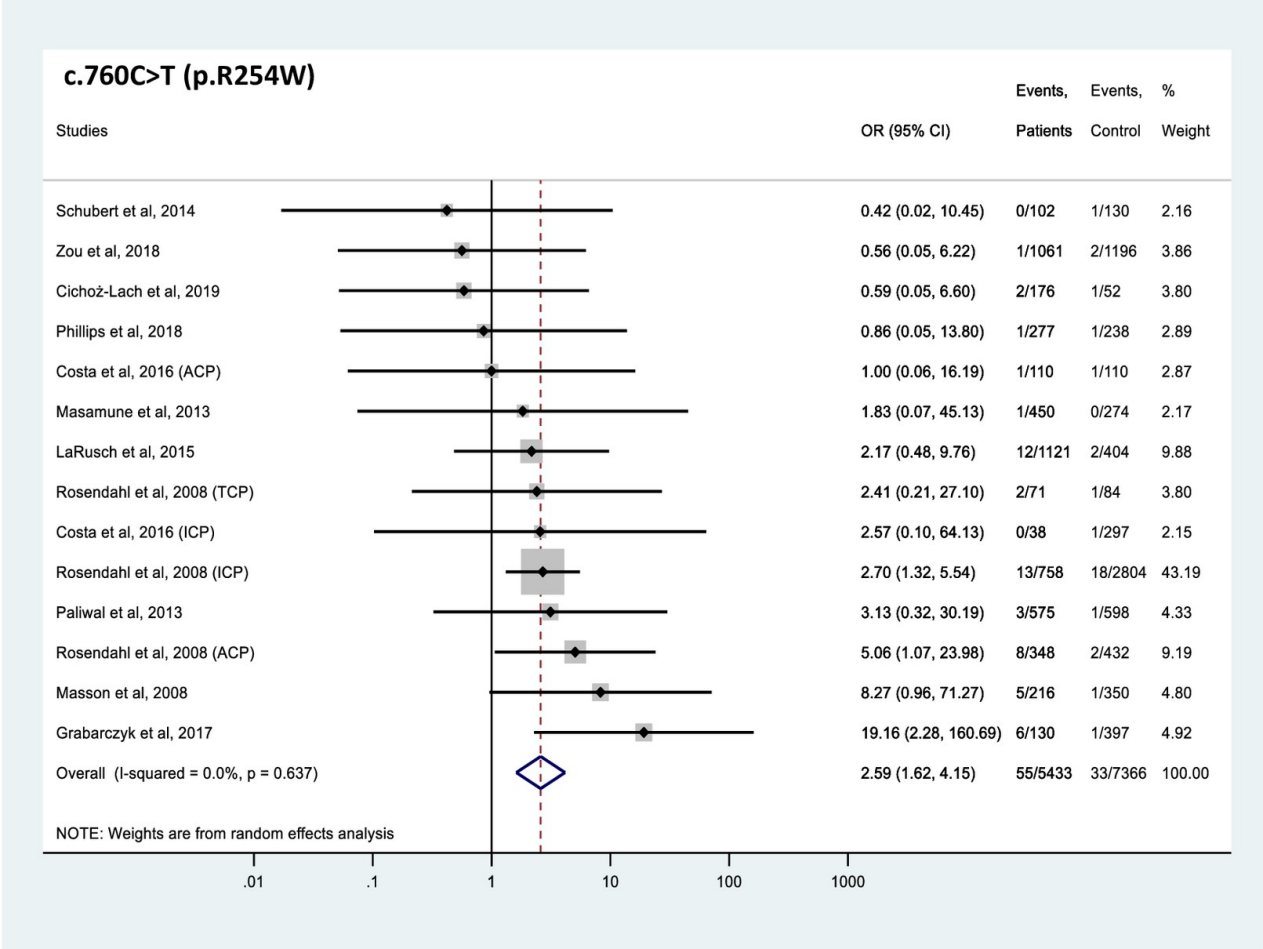
Forest plot showing odds ratios for pancreatitis risk in patients carrying the c.760C>T (p.R254W) *CTRC* variant. OR, odds ratio; CI, confidence interval.

All 4 *CTRC* variants analyzed were found significantly more frequently in patients with CP than in controls (Figs 2–5). The effect sizes, as judged by the OR values, were similar for the 4 variants and ranged from 2.6 to 6.5. Comparable OR values were calculated for variants p.V235I (OR 4.5, 95% CI 2.2–9.1), and p.K247_R254del (OR 5.4, 95% CI 2.6–11.0) while variant p.A73T (OR 6.5, 95% CI 2.4–17.8) was associated with slightly higher and variant p.R254W (OR 2.6, 95% CI 1.6–4.2) with slightly lower risk. Global carrier frequencies for variants p.A73T, p.V235I, p.K247_R254del, and p.R254W in CP were 2.4%, 1.2%, 1.1% and 1.0%, whereas the highest reported carrier frequencies in CP were 5.6%, 4.9%, 5.3% and 4.6%, respectively.

The vast majority of carriers had heterozygous *CTRC* variants, whereas homozygous and compound heterozygous cases were rare. When we considered all homozygous and compound heterozygous *CTRC* variant carriers together (n = 13) versus controls, the meta-analysis yielded an OR value of 10.8 (95% CI 2.4–49.6), indicating a higher risk effect than seen with any of the heterozygous *CTRC* variants (S1 Fig). However, the small event number and the arbitrary pooling of variants limits the usefulness of this estimate.

To address the role of *CTRC* variants in alcoholic CP, we performed a meta-analysis for variants p.K247_R254del (S2 Fig) and p.R254W (S3 Fig), which were detected in association with alcoholic CP in a handful of studies. No data were available for variants p.A73T and p.V235I. For this calculation, we compared alcoholic CP cases versus healthy controls. We confirmed disease association of variants p.K247_R254del (OR 5.4, 95% CI 1.4–21.5) and p.R254W (OR 2.4, 95% CI 1.2–4.6) with effect sizes that were similar to those seen in the overall CP group.

### Linkage disequilibrium between variant p.K247_R254del and the common c.180C>T (p.G60 =) haplotype

During our review of unpublished *CTRC* sequencing data from the Hungarian Pancreatic Study Group, we noted that three patients carrying a heterozygous microdeletion variant were also positive for the common p.G60 = variant; including 2 homozygous cases (Table 2). To explore whether these two variants are in linkage disequilibrium, we re-analyzed genetic data of 12 subjects of German origin from Rosendahl, et al. (2008) and 11 individuals of Polish origin from Grabarczyk, et al. (2017). Two unpublished cases of Turkish and Serbian origin sequenced in Germany were also included. Taken together, from the 28 subjects evaluated, 27 carried both variants, and in 9 cases the p.G60 = variant was homozygous.

**Table 2:** Association of the *CTRC* c.738_761del24 (p.K247_R254del) microdeletion with the common *CTRC* variant c.180C>T (p.G60 =).

Country	Etiology	Gender	Age of onset	p.K247_R254del	p.G60 =	Reference
Germany	ACP	M	48	hetero	hetero	Rosendahl, 2008
Germany	NACP	M	23	hetero	hetero	Rosendahl, 2008
Germany	NACP	M	12	hetero	hetero	Rosendahl, 2008
Germany	NACP	F	1	hetero	**homo**	Rosendahl, 2008
Germany	NACP	F	28	hetero	**homo**	Rosendahl, 2008
Germany	NACP	M	10	hetero	hetero	Rosendahl, 2008
Germany	NACP	M	24	hetero	hetero	Rosendahl, 2008
Germany	NACP	M	45	hetero	hetero	Rosendahl, 2008
Germany	NACP	F	49	hetero	hetero	Rosendahl, 2008
Austria	NACP	F	7	hetero	hetero	Rosendahl, 2008
Poland	NACP	M	10	hetero	hetero	Grabarczyk, 2017
Poland	NACP	F	5	hetero	hetero	Grabarczyk, 2017
Poland	NACP	F	13	hetero	**homo**	Grabarczyk, 2017
Poland	NACP	M	4	hetero	**homo**	Grabarczyk, 2017
Poland	NACP	M	4	hetero	hetero	Grabarczyk, 2017
Poland	NACP	F	7	hetero	**homo**	Grabarczyk, 2017
Poland	NACP	M	7	hetero	**homo**	Grabarczyk, 2017
Turkey	NACP	F	31	hetero	hetero	unpublished
Serbia	NACP	M	26	hetero	**homo**	unpublished
Hungary	NACP	M	20	hetero	**homo**	unpublished
Hungary	NACP	M	9	hetero	**homo**	unpublished
Hungary	NACP	M	18	hetero	hetero	unpublished
Germany	alcoholic control	M	n/a	hetero	hetero	Rosendahl, 2008
Germany	control	F	n/a	hetero	hetero	Rosendahl, 2008
Poland	control	n/a	n/a	hetero	hetero	Grabarczyk, 2017
Poland	control	n/a	n/a	hetero	hetero	Grabarczyk, 2017
Poland	control	n/a	n/a	hetero	**-**	Grabarczyk, 2017
Poland	control	n/a	n/a	hetero	hetero	Grabarczyk, 2017

ACP, alcoholic chronic pancreatitis; NACP, non-alcoholic chronic pancreatitis; RAP, recurrent acute pancreatitis. Hetero, heterozygous carrier; homo, homozygous carrier.

### Quality assessment and publication bias

No heterogeneity was observed. Sensitivity analysis (leave-one-out method) revealed no significant impact of any given study on the summary OR values (S4 Fig). Results of the modified Newcastle-Ottawa Scale are shown in S2 Table. With a single exception, all studies met the high-quality criteria. Funnel plots were generated for the p.V235I and p.R254W variants. Visual assessment and Egger’s test did not indicate a publication bias (S5 Fig).

## Discussion

CP is a progressive fibro-inflammatory disorder of the pancreas characterized by acinar cell atrophy with diffuse fibrosis, inflammatory cell infiltrates, regenerative pseudotubular complexes, and ductal irregularities with calcifications [1]. Progressive fibrosis is thought to be responsible for the development of the long-term clinical sequelae such as chronic pain, exocrine insufficiency and diabetes.

The aim of the present study was to obtain a formal estimate of the risk of CP in carriers of loss-of-function *CTRC* variants. To this end, we performed a meta-analysis of published studies that assessed the distribution of four low-frequency variants (>1%) between patients and controls. We chose these four variants, including three missense mutations and a microdeletion, because their genetic association with CP has been reproducibly documented and experimental studies confirmed the variants caused loss of CTRC function. Thus, the effect size of these variants on CP risk should be generally applicable to other rare or private *CTRC* missense mutations as well. The commonly occurring *CTRC* risk variant c.180C>T (p.G60 =) was excluded from this study and will be analyzed elsewhere, because it does not alter the CTRC amino-acid sequence, and its functional impact on CTRC has not been clarified yet.

Considering the relatively small number of publications suitable for meta-analysis, the lack of standardized cohort definitions with respect to etiology, and the variable reporting of homozygous and compound heterozygous carriers, we chose to perform a “global” analysis in which all CP cohorts were included and carrier frequencies (rather than alleles or genotypes) were considered. We note, however, that this global CP cohort mostly represents heterozygous *CTRC* variant carriers with non-alcoholic CP. Using this approach, the meta-analysis revealed that *CTRC* variants increased CP risk by about 3–7 fold, as estimated by OR values. Homozygous or compound heterozygous carriers were rare but seemed to increase risk more significantly, by about 11-fold, which we consider a lower-end estimate. Subgroup analysis of alcoholic CP cases indicated a similar risk increase as seen in the global CP cohort. Recently, *Chen et al*. (2021) investigated gene-alcohol interactions in CP and reported higher risk in case of the c.760C>T (p.R254W) variant in alcoholic (OR = 2.87) versus non-alcoholic CP (OR = 1.98). We believe this may be a spurious finding due to the smaller case numbers analyzed than in our study. Importantly, in the same article, the cumulative analysis of rare pathogenic *CTRC* variants in exons 2,3 and 7 showed no difference between the alcoholic and non-alcoholic CP groups (OR = 4.25 and 4.05, respectively), supporting the notion that *CTRC* variants contribute comparable risk effects to these two CP etiologies [26]. In contrast to *CTRC*, variants in *SPINK1* have a smaller impact in alcoholic than in non-alcoholic CP while gain-of-function *PRSS1* variants are almost never found in alcoholic disease [2, 26].

The functional consequences of variants p.A73T, p.V235I, p.K247_R254del, and p.R245W were previously characterized in cell culture experiments with transfected HEK 293T cells and adenovirus-transduced AR42J cells [4, 21, 23]. In addition, enzymatic activity of CTRC variants was tested using purified CTRC protein. Based on these assays, variants p.A73T and p.K247_R254del were classified as high-risk variants and variants p.V235I and p.R245W as moderate-to-low risk variants [21]. Variant p.K247_R254del was found to exhibit a complete loss of function as it had no protease activity whatsoever and trypsin rapidly degraded it. Similarly, nearly complete (80–90%) loss of function was observed with variant p.A73T, which was poorly secreted from cells. Furthermore, variant p.A73T induced endoplasmic reticulum (ER) stress indicating that mutation-induced misfolding and intracellular retention might explain its defective secretion. It remains unclear whether ER stress would contribute to CP risk in carriers of variant p.A73T. In contrast to variants p.A73T and p.K247_R254del, we found that the functional defect in variants p.V235I and p.R245W was less conspicuous. Variant p.V235I was secreted almost as well as wild-type CTRC from HEK 293T cells and the purified protein had circa 70% enzyme activity on a small peptide substrate. However, when this variant was tested in trypsinogen degradation experiments, it exhibited only about 50% activity relative to wild-type CTRC. Variant p.R254W was secreted to reduced levels (50% of wild type) from HEK 293T cells, while secretion from AR42J cells was almost normal (80%). The purified p.R254W protein was fully active on a small peptide substrate but its trypsinogen-degrading ability has not been tested so far. Variant p.R254W was also prone to degradation by high concentrations of trypsin. Taken together, available evidence indicates that variant p.V235I causes an about 50% loss of function, while the extent of the functional impairment in variant p.R254W remains hard to define but appears relatively small.

When the OR values are compared with the functional properties of the mutants, the difference between the high-risk *CTRC* variants p.A73T (OR 6.5) and p.K247_R254del (OR 5.4) versus the low-risk variant p.R254W (OR 2.6) is apparent and supports the validity of the results of our meta-analysis. Curiously, variant p.V235I (OR 4.5) is an outlier in this classification, as its risk effect would suggest a stronger functional defect than experimentally documented. We note, however, that the functional analysis published to date is limited in scope and variant p.V235I may cause loss of CTRC function by other mechanisms not tested yet, e.g. by affecting mRNA expression or splicing. Despite the imperfect correlation between the risk effects and the functional loss of *CTRC* variants, our current study establishes a solid baseline value for the effect size of heterozygous *CTRC* null variants, which is around 5-6-fold increased risk of CP. As noted above, homozygous and compound heterozygous *CTRC* variants are expected to impart substantially higher risk, however, available data are limited.

An unexpected “bonus” observation from this study is the linkage disequilibrium between the p.K247_R254del microdeletion variant and the common p.G60 = haplotype. Even though several laboratories reported both variants in their cohorts; their association has never been described before. Genetic counselors of patients with *CTRC* variants need to take this new information into consideration when determining the overall risk of CP. Although the p.G60 = haplotype increases CP risk about 2-fold, this effect will be negated by the presence of the microdeletion on the same allele and the overall risk will be solely determined by the heterozygous microdeletion variant. Furthermore, in case of a heterozygous microdeletion occurring together with p.G60 = homozygosity, one should consider only one p.G60 = allele when estimating compounded disease risk.

A limitation of the present study is the global nature of the analysis, which may mask higher or lower effect sizes associated with certain etiology, geography or demographics. In this regard, it is noteworthy that variants p.A73T and p.V235I are prevalent in India, while variants p.K247_R254del and p.R254W were predominantly reported from Europe. Restricting the analysis to Indian versus European cohorts might yield higher OR values for given variants than the global approach. Similarly, the impact of *CTRC* variants may be higher in pediatric CP versus adult-onset CP, as suggested by a Polish study [17]. Despite these unresolved questions, our meta-analysis offers strong support for the notion that *CTRC* variants are relatively strong risk factors for CP and argues for routine genetic screening of patients in the clinical setting.

## Supporting information

S1 Fig Forest plot showing odds ratios for pancreatitis risk in homozygous and compound heterozygous *CTRC* variant carriers. OR, odds ratio; CI, confidence interval. Two patients were homozygous for p.A73T, eight patients and one control for p.V235I. Compound heterozygosity was confirmed in three cases (p.A73T/p.V235I; p.V235I/p.R254W; and p.V235I/p.K247_R254del, respectively).
(PPTX)

S2 Fig Forest plot showing odds ratios for pancreatitis risk in patients with alcoholic chronic pancreatitis (ACP) carrying the c.738_761del24 (p.K247_R254del) *CTRC* variant. OR, odds ratio; CI, confidence interval.
(PPTX)

S3 Fig Forest plot showing odds ratios for pancreatitis risk in patients with alcoholic chronic pancreatitis (ACP) carrying the c.760C>T (p.R254W) *CTRC* variant. OR, odds ratio; CI, confidence interval.
(PPTX)

S4 Fig Sensitivity analyses (leave-one-out) of the studies included in the meta-analysis. **A,** c.217G>A (p.A73T); **B,** c.703G>A (p.V235I); **C,** c.738_761del24 (p.K247_R254del); **D,** c.760C>T (p.R254W). No significant impact of any given study on the summary OR values has been found.
(PPTX)

S5 Fig Funnel plots evaluating the effect of publication bias. **A,** c.703G>A (p.V235I); **B,** c.760C>T (p.R254W). Visual assessment and Egger’s test did not indicate a publication bias.
(PPTX)

S1 Table PRISMA 2020 checklist. (PDF)

S2 Table Newcastle-Ottawa Scale (NOS) for quality assessment of the case-control studies selected for meta-analysis. Each study was evaluated using a star system based on three broad categories; selection of study subjects (cases and controls), comparability of subjects, and ascertainment of the exposure. The maximum score of the NOS was 8 points. Studies with a score ≥ 6 points were considered high quality. With a single exception, all studies met the high-quality criteria.
(DOCX)
