# Supplementary figures and images for: Risk of chronic pancreatitis in carriers of loss-of-function CTRC variants: A meta-analysis
Source: PLoS One. 2022 May 20;17(5):e0268859. doi: 10.1371/journal.pone.0268859 (PMC9122191; doi:10.1371/journal.pone.0268859)

## Slide 1
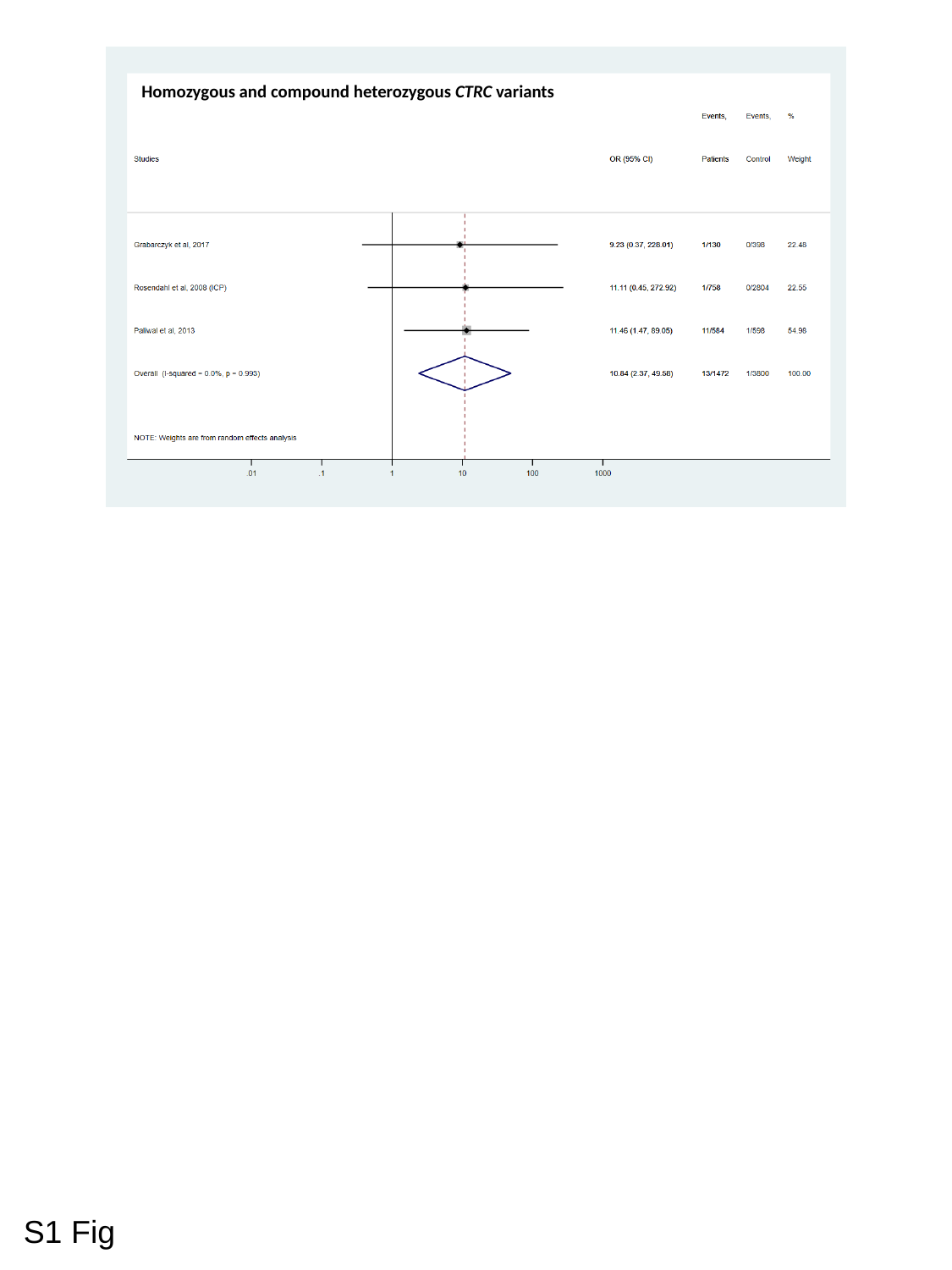

Homozygous and compound heterozygous CTRC variants
S1 Fig

Supplement: S1 Fig — OR, odds ratio; CI, confidence interval. Two patients were homozygous for p.A73T, eight patients and one control for p.V235I. Compound heterozygosity was confirmed in three cases (p.A73T/p.V235I; p.V235I/p.R254W; and p.V235I/p.K247_R254del, respectively). (PPTX) [file pone.0268859.s001.pptx]

## Slide 1
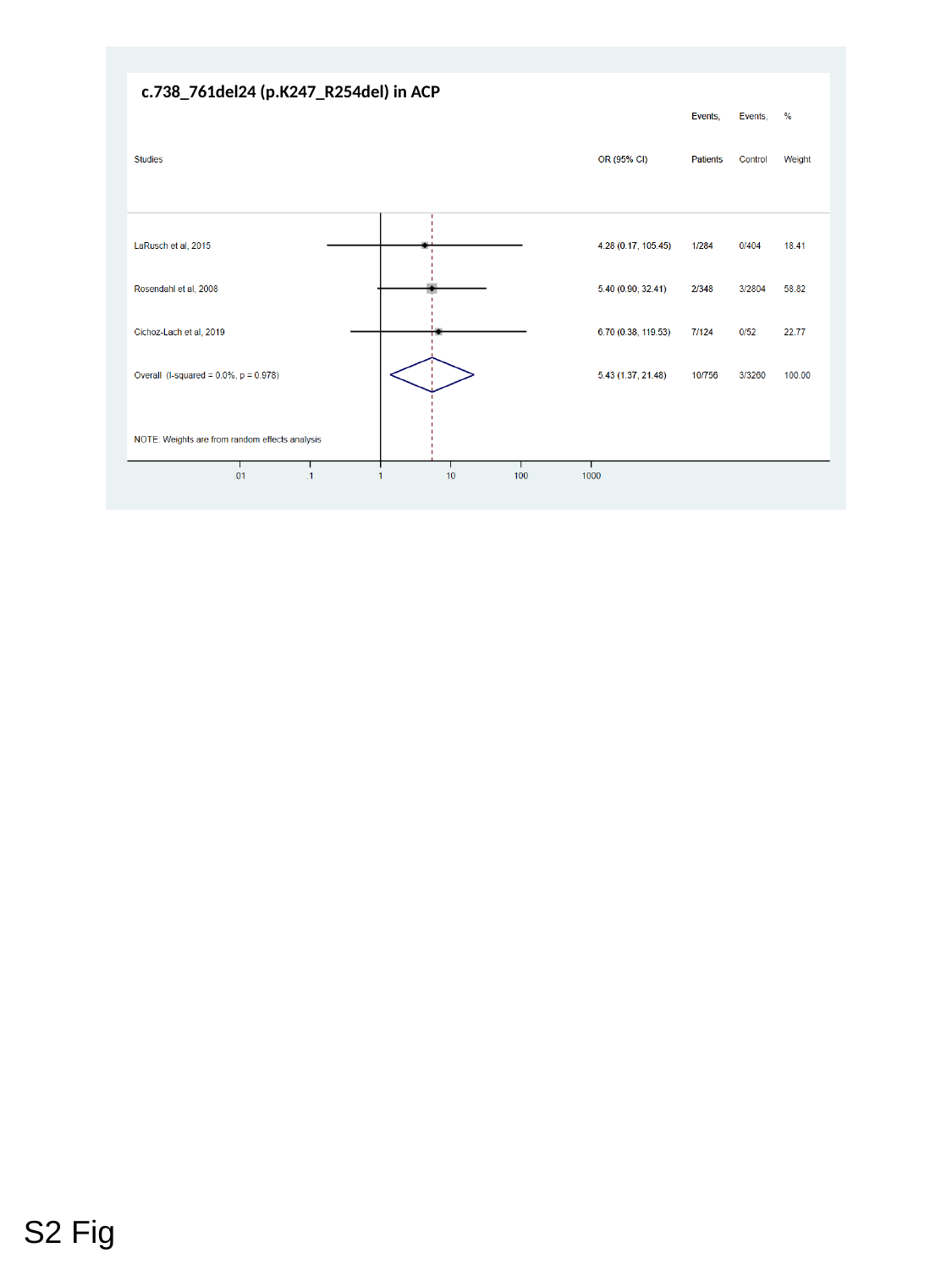

c.738_761del24 (p.K247_R254del) in ACP
S2 Fig

Supplement: S2 Fig — OR, odds ratio; CI, confidence interval. (PPTX) [file pone.0268859.s002.pptx]

## Slide 1
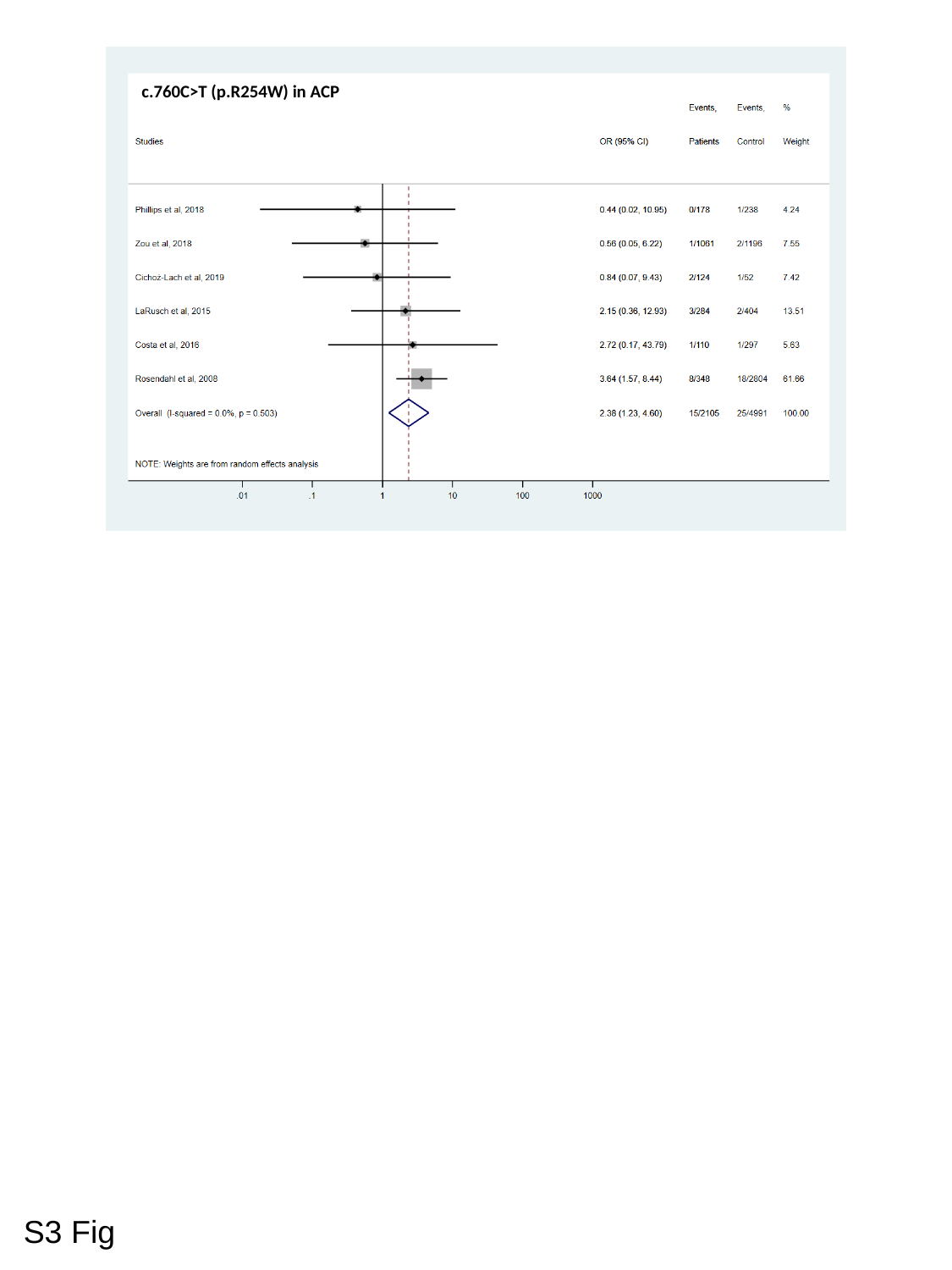

c.760C>T (p.R254W) in ACP
S3 Fig

Supplement: S3 Fig — OR, odds ratio; CI, confidence interval. (PPTX) [file pone.0268859.s003.pptx]

## Slide 1
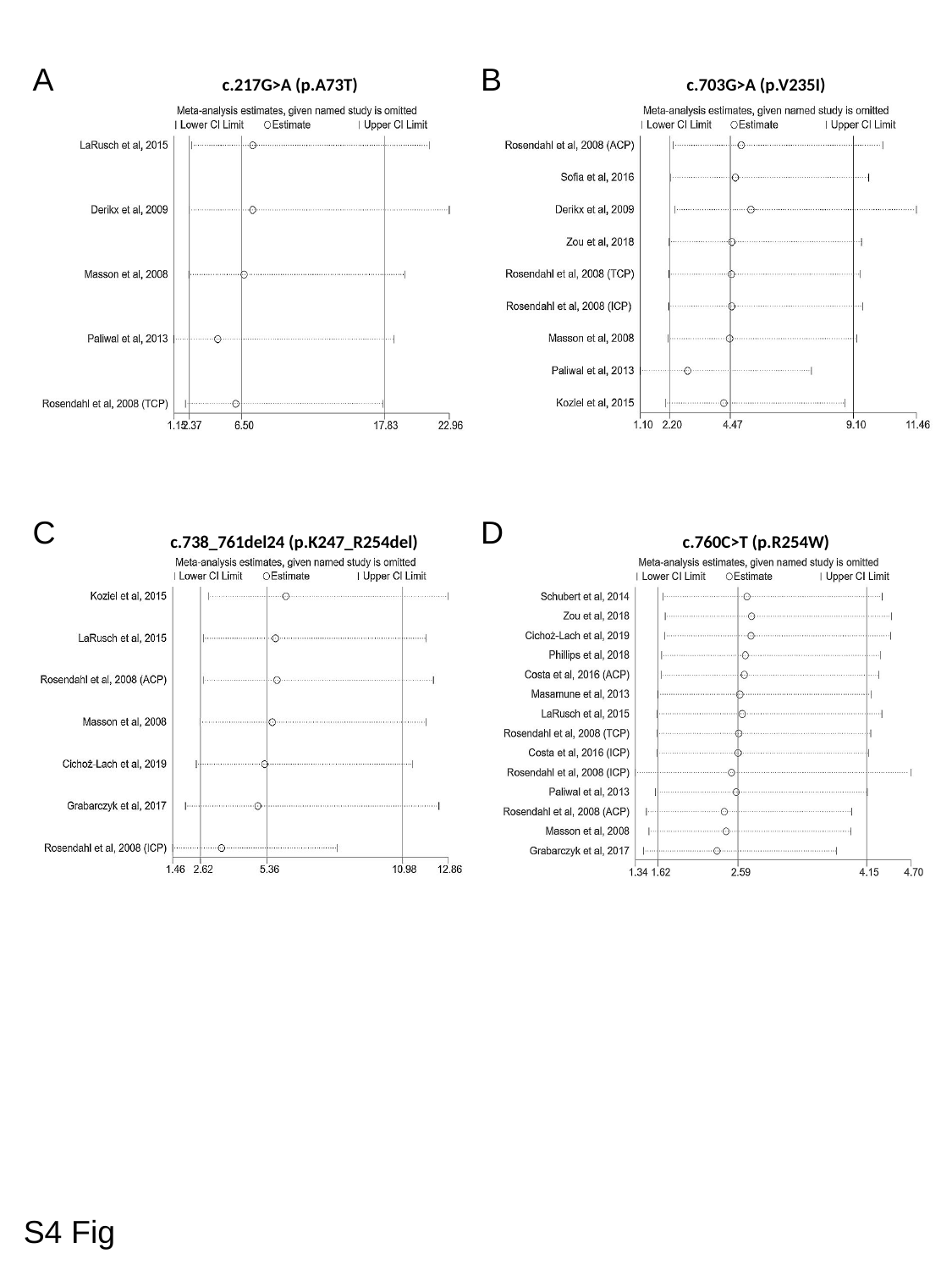

A
B
c.217G>A (p.A73T)
c.703G>A (p.V235I)
C
D
c.738_761del24 (p.K247_R254del)
c.760C>T (p.R254W)
S4 Fig

Supplement: S4 Fig — A, c.217G>A (p.A73T); B, c.703G>A (p.V235I); C, c.738_761del24 (p.K247_R254del); D, c.760C>T (p.R254W). No significant impact of any given study on the summary OR values has been found. (PPTX) [file pone.0268859.s004.pptx]

## Slide 1
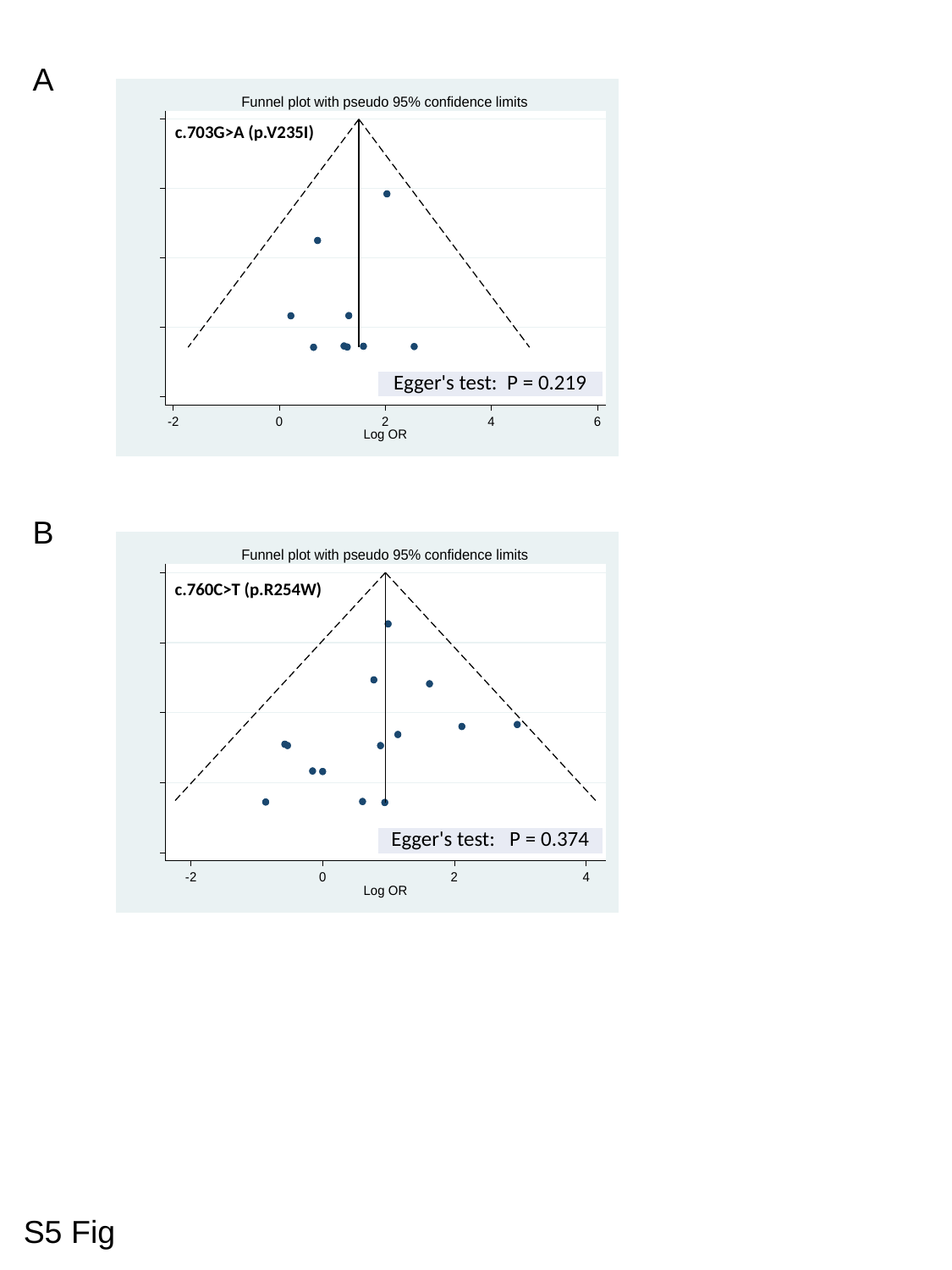

A
c.703G>A (p.V235I)
| Egger's test: P = 0.219 |
| --- |
B
c.760C>T (p.R254W)
| Egger's test: P = 0.374 |
| --- |
S5 Fig

Supplement: S5 Fig — A, c.703G>A (p.V235I); B, c.760C>T (p.R254W). Visual assessment and Egger’s test did not indicate a publication bias. (PPTX) [file pone.0268859.s005.pptx]
