# Supplementary material for: Risk of chronic pancreatitis in carriers of loss-of-function CTRC variants: A meta-analysis
Source: PLoS One. 2022 May 20;17(5):e0268859. doi: 10.1371/journal.pone.0268859 (PMC9122191; doi:10.1371/journal.pone.0268859)
Supplement: S2 Table — Each study was evaluated using a star system based on three broad categories; selection of study subjects (cases and controls), comparability of subjects, and ascertainment of the exposure. The maximum score of the NOS was 8 points. Studies with a score ≥ 6 points were considered high quality. With a single exception, all studies met the high-quality criteria. (DOCX) [file pone.0268859.s007.docx]

**S2 Table.** **Newcastle-Ottawa Scale (NOS) for quality assessment of the case-control studies selected for meta-analysis.** Each study is evaluated using a star system based on three broad categories; selection of study subjects (cases and controls), comparability of subjects, and ascertainment of the exposure. The maximum score of the NOS is 8 points. Studies with a score ≥ 6 points were considered high quality.

| **Study** | **Selection** | | | | **Comparability** | **Exposure** | |
| --- | --- | --- | --- | --- | --- | --- | --- |
|  | Is the case definition adequate? | Representativness of the cases | Selection  of controls | Definition of controls | Comparability of cases and controls based on design or analysis | Assessment of exposure | Same method of ascertainment for cases and controls |
| Rosendahl et al., 2008 | * | * | * | * | ** | * | * |
| Masson et al., 2008 | * | * | * | * | ** | * | - |
| Derikx et al., 2009 | * | * | - | - | ** | * | * |
| Paliwal et al., 2013 | * | * | * | * | ** | * | - |
| Masamune et al., 2013 | * | * | * | * | ** | ­* | * |
| Schubert et al., 2014 | * | * | * | * | ** | * | * |
| LaRusch et al., 2015 | * | * | * | * | ** | - | * |
| Koziel et al., 2015 | * | * | * | * | ** | - | * |
| Sofia et al., 2016 | * | - | * | * | ** | * | * |
| Costa et al., 2016 | * | * | * | * | ** | * | * |
| Grabarczyk et al., 2017 | * | * | * | * | ** | - | - |
| Phillips et al., 2018 | * | * | - | - | ** | - | ­* |
| Zou et al., 2018 | * | * | * | * | ** | * | * |
| Cichoż-Lach et al., 2019 | * | * | * | * | ** | * | * |
